# The combination of fluoxetine and environmental enrichment reduces postpartum stress-related behaviors through the oxytocinergic system and HPA axis in mice

**DOI:** 10.1038/s41598-021-87800-z

**Published:** 2021-04-19

**Authors:** Hamideh Bashiri, Danielle J. Houwing, Judith R. Homberg, Ali-Akbar Salari

**Affiliations:** 1 Neuroscience Research Center, Institute of Neuropharmacology, Department of Physiology and Pharmacology, Kerman University of Medical Sciences, Kerman, Iran; 2 Sirjan School of Medical Sciences, Sirjan, Iran; 3 Department of Cognitive Neuroscience, Center for Medical Neuroscience, Donders Institute for Brain, Cognition, and Behaviour, Radboud University Medical Center, Nijmegen, The Netherlands; 4 Salari Institute of Cognitive and Behavioral Disorders (SICBD), Karaj, Alborz, Iran

**Keywords:** Stress and resilience, Receptor pharmacology, Neuroscience, Anxiety, Depression

## Abstract

Gestational stress can increase postpartum depression in women. To treat maternal depression, fluoxetine (FLX) is most commonly prescribed. While FLX may be effective for the mother, at high doses it may have adverse effects on the fetus. As environmental enrichment (EE) can reduce maternal stress effects, we hypothesized that a subthreshold dose of FLX increases the impact of EE to reduce anxiety and depression-like behavior in postpartum dams exposed to gestational stress. We evaluated this hypothesis in mice and to assess underlying mechanisms we additionally measured hypothalamic–pituitary–adrenal (HPA) axis function and brain levels of the hormone oxytocin, which are thought to be implicated in postpartum depression. Gestational stress increased anxiety- and depression-like behavior in postpartum dams. This was accompanied by an increase in HPA axis function and a decrease in whole-brain oxytocin levels in dams. A combination of FLX and EE remediated the behavioral, HPA axis and oxytocin changes induced by gestational stress. Central administration of an oxytocin receptor antagonist prevented the remediating effect of FLX + EE, indicating that brain oxytocin contributes to the effect of FLX + EE. These findings suggest that oxytocin is causally involved in FLX + EE mediated remediation of postpartum stress-related behaviors, and HPA axis function in postpartum dams.

## Introduction

Maternal depression frequently occurs during pregnancy, with 1 in 5 women reporting depressive symptoms^1^. An estimated 3–7% of pregnant women suffer from a major depressive disorder^2–6^. Stress-related disorders such as depression during late pregnancy are a major risk factor for the development of postpartum depression^7^. Maternal depression during pregnancy and postpartum period has been associated with adverse effects on child neurodevelopment^8–13^.

During pregnancy and the postpartum period, the function of the mothers' hypothalamic pituitary adrenal (HPA) axis alters. In postpartum depression hypersecretion of cortisol has been observed^14,15^. Besides steroid hormones, the peptide hormone oxytocin (OXT) has recently gained attention for potential involvement in depression^16–19^. For instance, oxytocin was shown to be associated with the HPA axis function and depression in postpartum women, suggesting an interaction between oxytocin and glucocorticoids during the postpartum period on depressive behavior^20,21^.

To treat maternal depression, antidepressant selective serotonin reuptake inhibitors (SSRIs) are most commonly prescribed, with an international prevalence estimate of 3.0%^22^. Like maternal depression, using SSRIs during pregnancy can affect neurobehavioral development in the offspring^23–32^. In line with the human studies, rodent studies found that SSRI exposure during pregnancy and/or the postnatal period resulted in neurodevelopmental alterations in the offspring^33–50^. When the SSRI fluoxetine (FLX) is administered to dams^47,51–54^ its actions can markedly differ when using maternally stressed dams. Besides exerting beneficial effects, there might be risk that FLX treatment causes developmental changes in offspring, such as reduced activity and exploration behavior, an increased passive stress coping style, and less efficient sensory processing^55^. This asks for a more refined treatment regime effective for the mother and safe for the fetus. Environmental enrichment (EE) during pregnancy and postpartum period could also remediate anxiety and depression-like behaviors in mothers^56^. EE exposure affects HPA axis function by reducing stress hormones, including basal adrenocorticotropic hormone (ACTH) and corticosterone levels of singly housed male and female rats^57^. Furthermore, EE was shown to activate the oxytocinergic system in the brain of mice^58^. However, it is not known whether EE during pregnancy and postpartum period can prevent gestational stress-induced depression-like behavior in the postpartum dam.

Of interest, it has recently been proposed that serotonin increases neuroplasticity, and thereby sensitivity to environmental stimuli, negative stimuli as well as positive ones such as EE. In support of this hypothesis^59^ it has been demonstrated that SSRI treatment in combination with an enriched environment reduces depression-like behavior in mice^60,61^. As to whether these effects extend to maternal depression is up to date unknown. Since perinatal FLX exposure can reduce maternal stress effects, we questioned whether a subthreshold dose of FLX would increase the impact of EE to reduce maternal depression.

The aim of this study was to investigate the effects of gestational-postpartum treatment with a relatively low (5 mg/kg) dose of FLX and EE, both separately as well as combined, on gestational stress-induced postpartum anxiety and depression-like behavior in mouse dams. It was our first hypothesis that a low dose of FLX combined with EE remediates anxiety- and depression-like behavior in gestationally stressed dams. To investigate underlying mechanisms, we measured HPA axis function, and brain OXT levels. Because we found that gestational stress was associated with a decrease in whole-brain oxytocin levels, and a combination of FLX and EE remediated anxiety- and depression-like behavior as well as this hormonal change, it was our second hypothesis that OXT would be causally involved in the remediation effects of FLX plus EE. To test this hypothesis, we tested whether central infusion of an OXT antagonist would prevent the remediation of postpartum stress-related behavior in dams following FLX and EE.

## Results

### Experiment 1

To test our hypotheses we studied the effects of gestational stress, fluoxetine treatment and environmental enrichment on anxiety (open field test), depression-like behavior (social interaction and sucrose preference, forced swim test), HPA axis function, and OXT in the brain.

### Open field test

**Figure 1:**
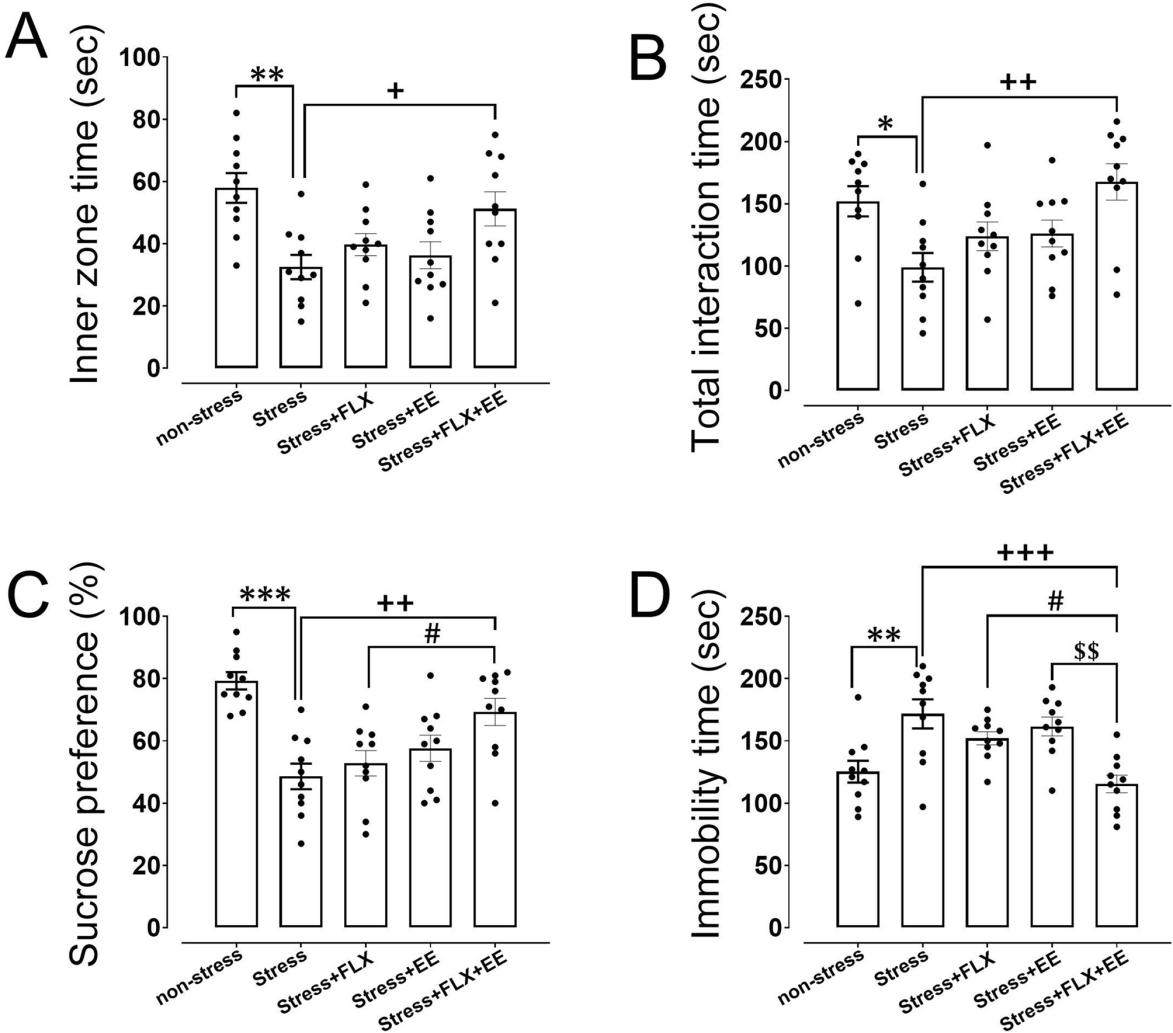
The effects of gestational stress, fluoxetine (FLX) treatment and environmental enrichment on anxiety-like behavior in the open field test (A; inner zone time) and depression-like behavior in the social interaction (B; total interaction time), sucrose preference (C), and forced swim test (D; immobility time) during the postpartum period in dams. Values are presented as mean ± SEM (N = 10). Significant differences: *P < 0.05, **P < 0.01 and ***P < 0.001, compared to non-stress group; + *P* < 0.05, +  + *P* < 0.01 and +  +  + *P* < 0.001, compared to stress group; #*P* < 0.05, compared to stress + FLX group; $$*P* < 0.01, compared to stress + EE group.

In the open field test the time spent in the inner zone was used as index of anxiety, which was significantly different between groups (F_(4,45)_ = 5.71, *p* = 0.002). Posthoc analysis revealed that gestational stress reduced the time spent in the inner zone of the open field compared to the non-stress condition, indicating that the gestational stress increased anxiety (*p* = 0.002; Fig. 1A). As expected, FLX treatment and EE independently could not restore stress-induced anxiety. Interestingly, a combination of FLX treatment and EE in stressed dams significantly increased the time spent in the inner zone compared to stressed only dams (*p* = 0.037; Fig. 1A), thus restoring anxiety levels back to normal.

### Social interaction test

For the total interaction time we found a significant difference between groups (F_(4,45)_ = 4.79, *p* = 0.004). Posthoc analysis demonstrated that gestational stress significantly decreased the total interaction time relative to the non-stress condition, leading to increased social withdrawal (*p* = 0.027; Fig. 1B). As expected, FLX treatment and EE independently did not restore stress-induced social withdrawal when compared to non-stress dams. However, a combination of FLX treatment and EE in stressed dams increased social interaction compared to stressed only dams (*p* = 0.002; Fig. 1B), indicating that social interaction was restored to non-stress level.

### Sucrose preference

We further observed that sucrose preference was significantly differed among groups (F_(4,45)_ = 10.14, *p* < 0.001). A subsequent analysis showed a significant reduction in sucrose preference in gestational stressed dams, when compared to non-stressed dams (*p* < 0.001; Fig. 1C). FLX treatment and EE independently did not restore stress-induced reductions in sucrose preference. However, FLX treatment and EE combined increased sucrose preference in stressed dams compared to stressed dams (*p* = 0.005; Fig. 1C) and stress + FLX dams (*p* = 0.038; Fig. 1C). This shows that the combination of FLX and EE reduced depression-like behavior.

### Forced swim test

To measure behavioral despair immobility time was measured, which was significantly different between groups (F_(4,45)_ = 8.27, *p* < 0.001). Posthoc analysis revealed that gestational stressed dams spent more time on immobility compared to non-stressed dams (*p* = 0.003; Fig. 1D), indicative for increased behavioral despair in stress dams. FLX treatment and EE independently did not reduce time spent on immobility. However, a combination of FLX treatment and EE in stressed dams reduced immobility time compared to stress dams (*p* < 0.001), stress + FLX dams (*p* = 0.026) and stress + EE dams (*p* = 0.003) (Fig. 1D). This finding suggests that combining FLX treatment and EE prevented stress-induced depression-like behavior.

### HPA axis function

**Figure 2:**
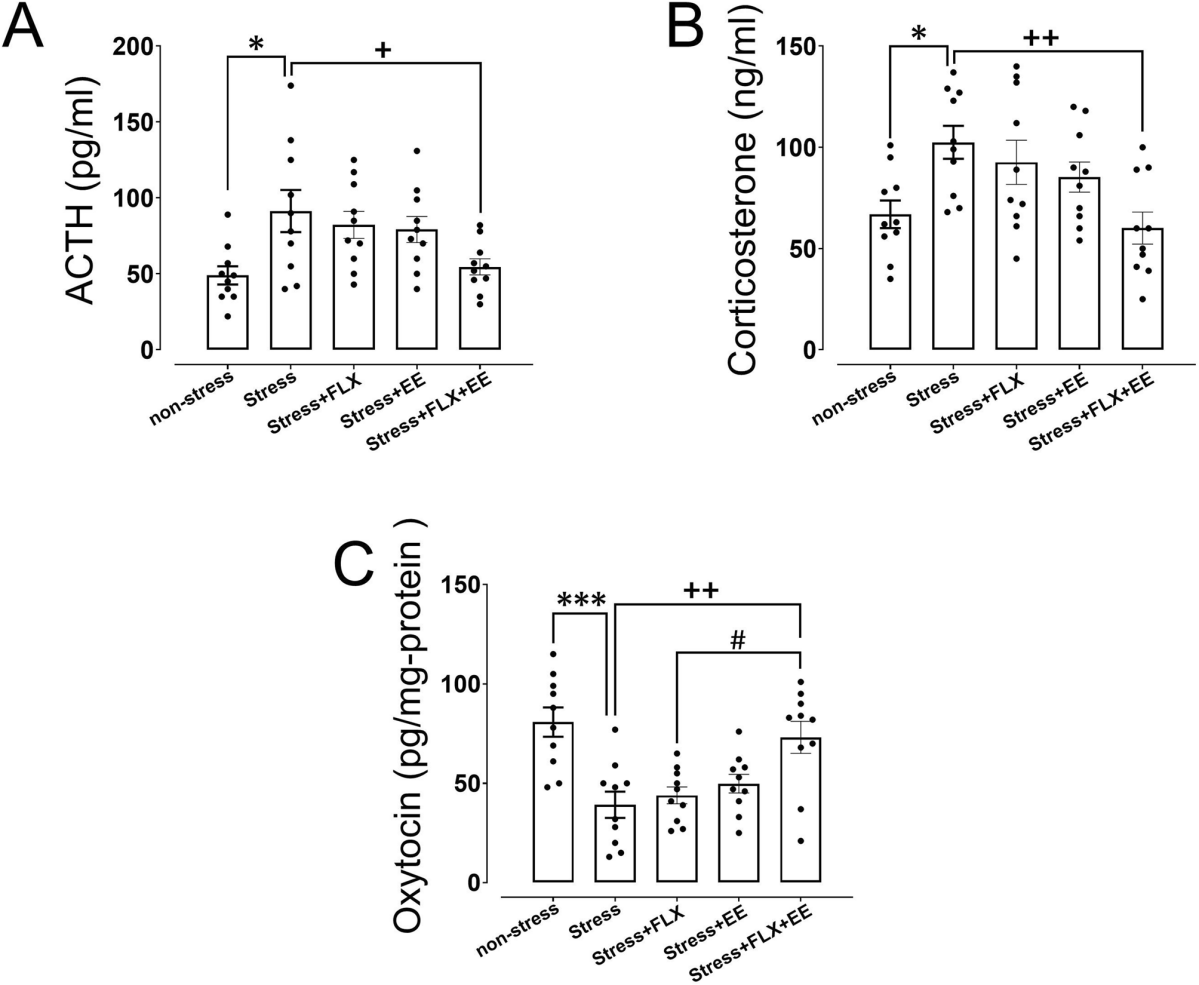
The effects of gestational stress, fluoxetine (FLX) treatment and environmental enrichment (EE) on ACTH (A) and corticosterone (B) in the serum, and oxytocin levels in the brain (C) during the postpartum period in dams. Values are presented as mean ± SEM (N = 10). Significant differences: *P < 0.05 and ***P < 0.001, compared to non-stress group; + *P* < 0.05 and +  + *P* < 0.01, compared to stress group; #*P* < 0.05, compared to stress + FLX group.

Both ACTH (F_(4,45)_ = 4.15, *p* = 0.006) and corticosterone (F_(4,45)_ = 4.46, p = 0.005) levels were between groups. As expected, a significant increase in ACTH (*p* = 0.015; Fig. 2A) and corticosterone (*p* = 0.033; Fig. 2B) levels was found in gestational stressed versus non-stressed postpartum dams. Moreover, the combination of FLX treatment and EE in stressed dams reduced ACTH (p = 0.046; Fig. 2A) and corticosterone (*p* = 0.007; Fig. 2B) levels compared to stress dams. FLX and EE did not independently reduce stress-induced ACTH and corticosterone levels. These findings demonstrate that a combination of FLX treatment and EE during pregnancy and lactation reduces HPA axis function to gestational stress during the postpartum period.

### Brain oxytocin

Lastly, brain OXT levels were significantly different between groups (F_(4,45)_ = 8.38, *p* < 0.001). Gestational stress significantly reduced OXT levels compared to dams left undisturbed (*p* < 0.001; Fig. 2C). The combination of FLX treatment and EE significantly increased OXT levels compared to both stressed dams (*p* = 0.004) and stress + FLX dams (*p* = 0.018; Fig. 2C). FLX treatment and EE independently did not increase OXT levels (*P* > 0.05).

### Experiment 2

As we observed in experiment 1 the FLX + EE mediated alleviation of the negative effects of gestational stress was associated with increased brain OXT levels, we tested in experiment 2 whether OXT receptor antagonist treatment could prevent the alleviating effects of FLX + EE. As readouts we focusing on anxiety (open field test) and depression-like behavior (social interaction, sucrose preference, forced swim test) and HPA axis function.

### Open field test

**Figure 3:**
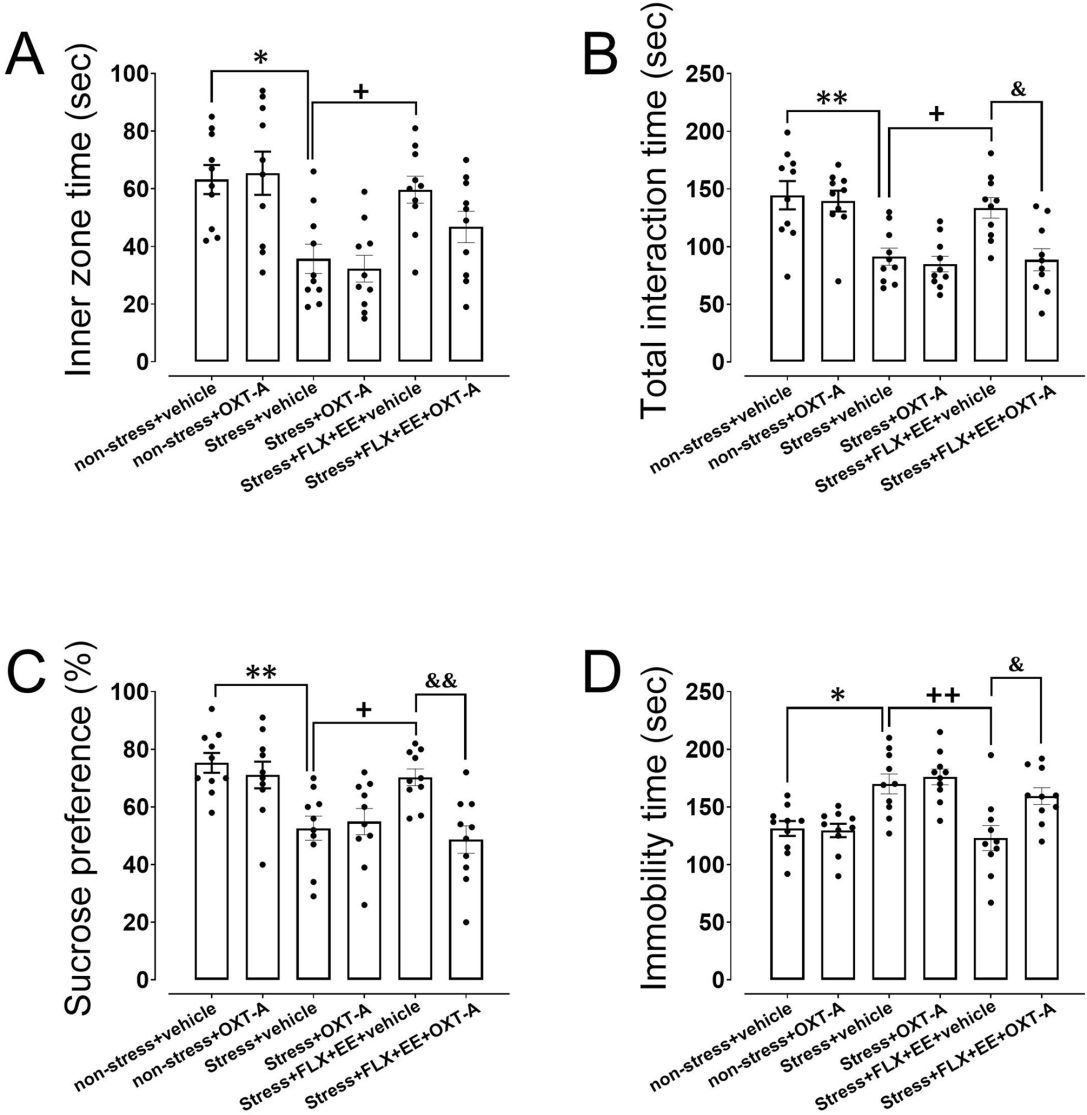
The effects of treatment with oxytocin receptor antagonist (OXT-A) following gestational stress, and fluoxetine treatment plus environmental enrichment (FLX + EE) on anxiety-like behavior in the open field test (A; inner zone time) and depression-like behavior in the social interaction (B; total interaction time), sucrose preference (C), and forced swim test (D; immobility time) during the postpartum period in dams. Values are presented as mean ± SEM (N = 10). Significant differences: *P < 0.05 and **P < 0.01, compared to non-stress group; + *P* < 0.05 and +  + *P* < 0.01, compared to stress group; &*P* < 0.05 and &&*P* < 0.01, compared to stress + FLX + EE + vehicle group.

One-way ANOVA revealed a significant difference between groups in time spent in the inner zone (F_(5,54)_ = 6.86, *p* < 0.001). As expected, posthoc analysis showed that gestational stress in dams significantly reduced the time spent in the inner zone, indicative for increased anxiety (*p* = 0.01; Fig. 3A). Furthermore, the combination of FLX treatment and EE significantly increased inner zone time compared to stressed only dams (*p* = 0.035; Fig. 3A). These findings confirm our results from experiment 1. No effects of OXT-A were observed in any of the groups when compared to vehicle-treated counterparts.

## Social interaction test

Regarding total time spent on social interaction we observed a significant difference between groups (F_(5,54)_ = 9.51, *p* < 0.001). Posthoc analysis revealed a significant reduction in total interaction time in gestational stressed compared to non-stressed dams (*p* = 0.002; Fig. 3B). In line with our findings from experiment 1, combined FLX treatment and EE significantly increased stress-induced total interaction time (*p* = 0.022; Fig. 3B). When OXT-A was administered to stressed dams that received combined FLX treatment and EE, the total interaction time significantly decreased compared to vehicle treated counterparts (*p* = 0.012; Fig. 3B). No effects of OXT-A were observed in non-stress and stress dams when compared to vehicle-treated counterparts. These results indicate that OXT-A mediated the effects of combined FLX treatment and EE on stress-induced social withdrawal.

### Sucrose preference

Sucrose preference was also significantly different between groups (F_(5,54)_ = 7.53, *p* < 0.001). As expected, gestational stress reduced sucrose preference compared to non-stress dams (*p* = 0.004; Fig. 3C). In addition, the combination of FLX treatment and EE significantly increased sucrose preference in stressed compared to non-stressed dams (*p* = 0.042; Fig. 3C), confirming our results from experiment 1. Interestingly, OXT-A prevented the increase in sucrose preference that was observed after combined FLX treatment and EE in stress dams (*p* = 0.007; Fig. 3C), while no effects of OXT-A were observed in non-stress and stress dams when compared to vehicle-treated counterparts. These results indicate that OXT-A mediated the effects of combined FLX treatment and EE on stress-induced alterations in anhedonia-like behavior.

### Forced swim test

The significant group differences in time spent on immobility (F_(5,54)_ = 8.58, *p* < 0.001), a measure of behavioral despair, were followed-up by posthoc analyses showed that gestational stress significantly increased immobility time relative to the non-stress condition (*p* = 0.012; Fig. 3D). The combination of FLX treatment and EE reduced the stress-induced increase in immobility time (*p* = 0.001; Fig. 3D). OXT-A prevented the effects of combined FLX treatment and EE on stress-induced immobility time (*p* = 0.021; Fig. 3D), while no effects of OXT-A were observed in non-stress and stress dams when compared to vehicle-treated counterparts. These results demonstrate that OXT plays a role in FLX + EE effects on stress-induced behavioral despair.

### HPA axis function

**Figure 4:**
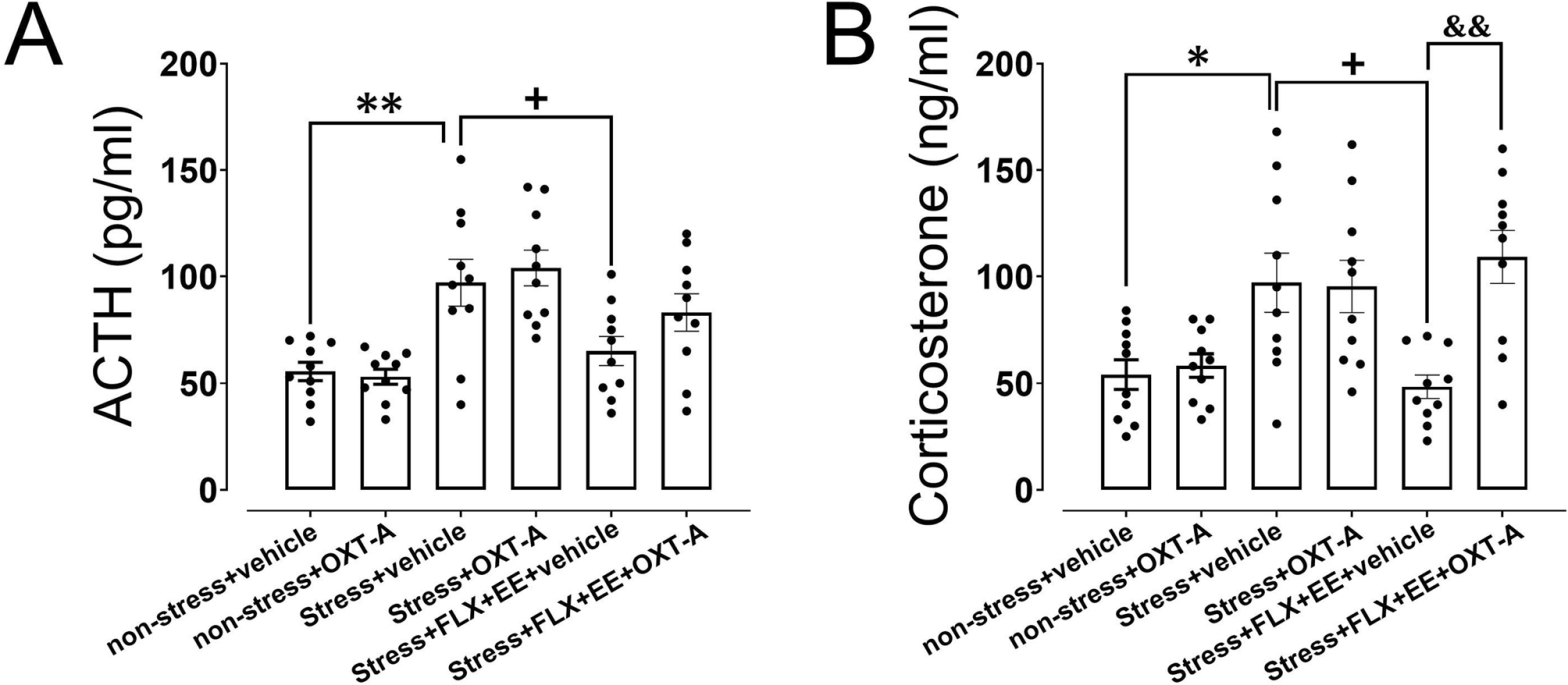
The effects of treatment with oxytocin receptor antagonist (OXT-A) following gestational stress, and fluoxetine treatment plus environmental enrichment (FLX + EE) on ACTH (A) and corticosterone (B) in the serum during the postpartum period in dams. Values are presented as mean ± SEM (N = 10). Significant differences: *P < 0.05 and **P < 0.01, compared to non-stress group; + *P* < 0.05, compared to stress group; &&*P* < 0.01, compared to stress + FLX + EE + vehicle group.

Lastly, we analyzed plasma ACTH and corticosterone levels. Group differences in ATCH (F_(5,54)_ = 8.11, *p* < 0.001) and corticosterone (F_(5,54)_ = 6.85, *p* < 0.001) levels were explained by a significant increase in both ACTH (*p* = 0.004; Fig. 4A) and corticosterone (*p* = 0.042; Fig. 4B) levels after gestational stress. Furthermore, the combination of FLX treatment and EE significantly reduced ACTH (*p* = 0.047; Fig. 4A) and corticosterone (*p* = 0.014; Fig. 4B) levels, showing preventive effects of FLX + EE on stress-induced changes in HPA-axis activity. OXT-A administration in stressed dams exposed to FLX treatment and EE significantly increased corticosterone –but not ACTH- levels compared to vehicle treated counterparts (*p* = 0.001; Fig. 4B). No effects of OXT-A were observed in non-stress and stress dams compared to vehicle treated dams. These findings demonstrate that OXT mediated the FLX + EE effects on stress-induced HPA-axis function.

## Discussion

In line with our hypothesis, we found that stress applied during pregnancy increased anxiety- and depression-like behavior in postpartum dams. This was accompanied by an increase in HPA axis function and an unexpected decrease in OXT levels in the dam. Further, as we hypothesized, a combination of subthreshold FLX and non-significant EE remediated the behavioral, HPA axis and OXT changes induced by gestational stress in postpartum dams. Central administration of an OXT receptor antagonist prevented the remediating effect of subthreshold FLX + non-significant EE, showing that brain OXT contributes to the combined effect of FLX and EE. The finding that gestational stress induced anxiety- and depression-like behavior in postpartum dams is in line with previous findings and suggests that stress during pregnancy can still exert its effects during the early postnatal period, when stress is absent^51,62^. This comes as no surprise, as maternal anxiety and depression during pregnancy is strongly associated with postpartum depressive symptoms in humans^63^.

Previous work from our group shows that perinatal FLX treatment with a higher dose (8 mg/kg) prevents depression-like behavior in postpartum dams^51^, while others show that effects of pre-gestational stress on serum corticosterone levels in postpartum dams can be reversed by perinatal treatment with 10 mg/kg FLX^64^. To date, studies directly investigating the effects of EE during pregnancy on gestationally stressed dams are lacking. Sparling et al. showed that the behavior of dams can be beneficially affected by a combination of physical and social enrichment during the gestational and postpartum periods^65^. However, rat dams housed in an enriched condition during their pregnancy and the postpartum period did not display any differences in anxiety-and depression-related symptoms compared to the control dams^65–67^. Most of the beneficial effects are exerted by EE consisting of increased cage complexity (larger cage, adding objects/shelters and a running wheel) and/or social enrichment. In the present study, we only increased cage complexity, which was by itself not strong enough to alter maternal anxiety- and depression-like behavior. We show, in line with the hypothesis that serotonin increase susceptibility to environmental stimuli such as EE^59^, that altering serotonin levels through subthreshold FLX treatment may enhance neuronal plasticity, rendering it more susceptible to the beneficial effects of non-significant EE.

Changes in HPA axis function are important mediators of the effects of gestational stress on postpartum anxiety- and depression-like behavior. We have previously shown that gestational stress can increase basal and stress-induced corticosterone levels in postpartum dams^51^. However, others have found that when the stress is pre-gestational, basal corticosterone levels were reduced postpartum and restored by perinatal FLX treatment^64^. These findings show that the timing of the stress and FLX treatment of the dams may differentially impact maternal corticosterone levels. We further observed that high corticosterone levels were correlated with increased forced swim test immobility and less social interaction. These results are in line with human findings, where a correlation between postpartum depression and abnormal HPA axis function and hypersecretion of cortisol is well-established^14,15^. Although studies have shown that EE can reduce HPA axis function by decreasing basal ACTH and corticosterone levels in rodents^68,69^, there are no reports studying the effect of EE on HPA axis activity in pregnant and postpartum dams. What makes our findings important is that we show that a combination of FLX and EE could reduce HPA axis function in dams. Further studies are needed to focus on different protocols of EE on HPA axis function during pregnancy and the postpartum period.

We further found that the gestational stress-induced anxiety and depression-like symptoms were paralleled by a decreased in whole-brain OXT levels. Interestingly, FLX + EE was related to decreased anxiety and depression-like behaviors and increased brain OXT levels. Brain OXT is one of the most important regulators of anxiety and depression in both humans and rodents^70^. OXT exerts anxiolytic and antidepressant effects and modulates neuronal functions associated with stress responses^70^. For example, intranasal OXT treatment was shown to decrease HPA axis activity, anxiety- and depression-related symptoms^71–73^. Since there is a significant comorbidity between these disorders^74,75^, common mediators such as OXT are presumably to underlie both psychiatric disorders^70^. In line with these studies, recent clinical and experimental researches have revealed that OXT treatment could be an interesting therapeutic strategy for postpartum depression^16^.

To investigate whether brain OXT is causally related to the remediating effects of FLX + EE we tested the effects of OXT-A treatment on anxiety- and depression-like behavior as well as HPA-axis function. Treatment with OXT-A did not further increase ACTH and corticosterone or behavioral measures in dams that were stressed during pregnancy, but did prevent the remediation of stress-induced effects by perinatal FLX treatment and EE. These findings indicate that OXT is causally involved in FLX + EE mediated remediation of stress-induced effects on HPA-axis function and anxiety- and depression-like behavior. In support of our findings, it has been recognized that EE exposure enhanced oxytocinergic system in the brain^58,76^. Besides, no significant changes were found following chronic treatment with SSRIs such as fluoxetine in basal oxytocin levels^77^. Interestingly, Cox et al., reported that the OXT surge during breastfeeding in women with or without anxiety and depression symptoms buffers stress-induced corticosterone secretion. They also found that postpartum depressed women had lower OXT levels during feeding than women without depression^21^. The present study shows that a significant increase of brain OXT levels following FLX + EE exposure could conceivably decrease anxiety and depression-related behaviors by reducing HPA axis function in stressed postpartum dams.

A unique aspect of this study that the behavior and hormonal state of dams was studied after exposure to various combinations of stress, FLX treatment and EE exposure. A notable limitation of is that we did not measure maternal behavior. Although the litters were culled to 8 mice (4 males, 4 females), we cannot exclude the possibility that changes in mother–pup interactions affected mother behavior and accordingly influenced the behavioral measurements in the dams. Taken together, our findings confirm and extend earlier research demonstrating OXT as an important player in the regulation of anxiety and depression during the postpartum period.

## Methods

### Animals

A total of 110 timed-pregnant NMRI mice (12–13 weeks old; 22–25 g) were produced in the animal facility at “Salari Institute of Cognitive and Behavioral Disorders (SICBD)”. All the experiments were performed in SICBD. The breeding procedure was performed as previously described^78^. To expose the female mice to male pheromones, two male and four female mice were placed in a partitioned cage with no physical contact for 3 days. In the next step, male and female mice were put in a cage one-by-one. The next morning (7:00 A.M.), the presence of a vaginal plug was considered a successful mating and gestational day (GD 0). Pregnant mice were housed individually in enriched or standard polycarbonate cages under standard laboratory conditions (12:12 h light/dark cycle; lights on 7:00 a.m.; temperature 23 ± 1 °C; humidity 40–50%). Food and water were available ad libitum. The experiments were approved by the Research and Ethics Committee of Kerman University of Medical Sciences (IR.KMU.REC.1399.325) and were in compliance with ARRIVE guidelines. All methods were also performed in accordance with the relevant guidelines and regulations.

### Experimental design

**Figure 5:**
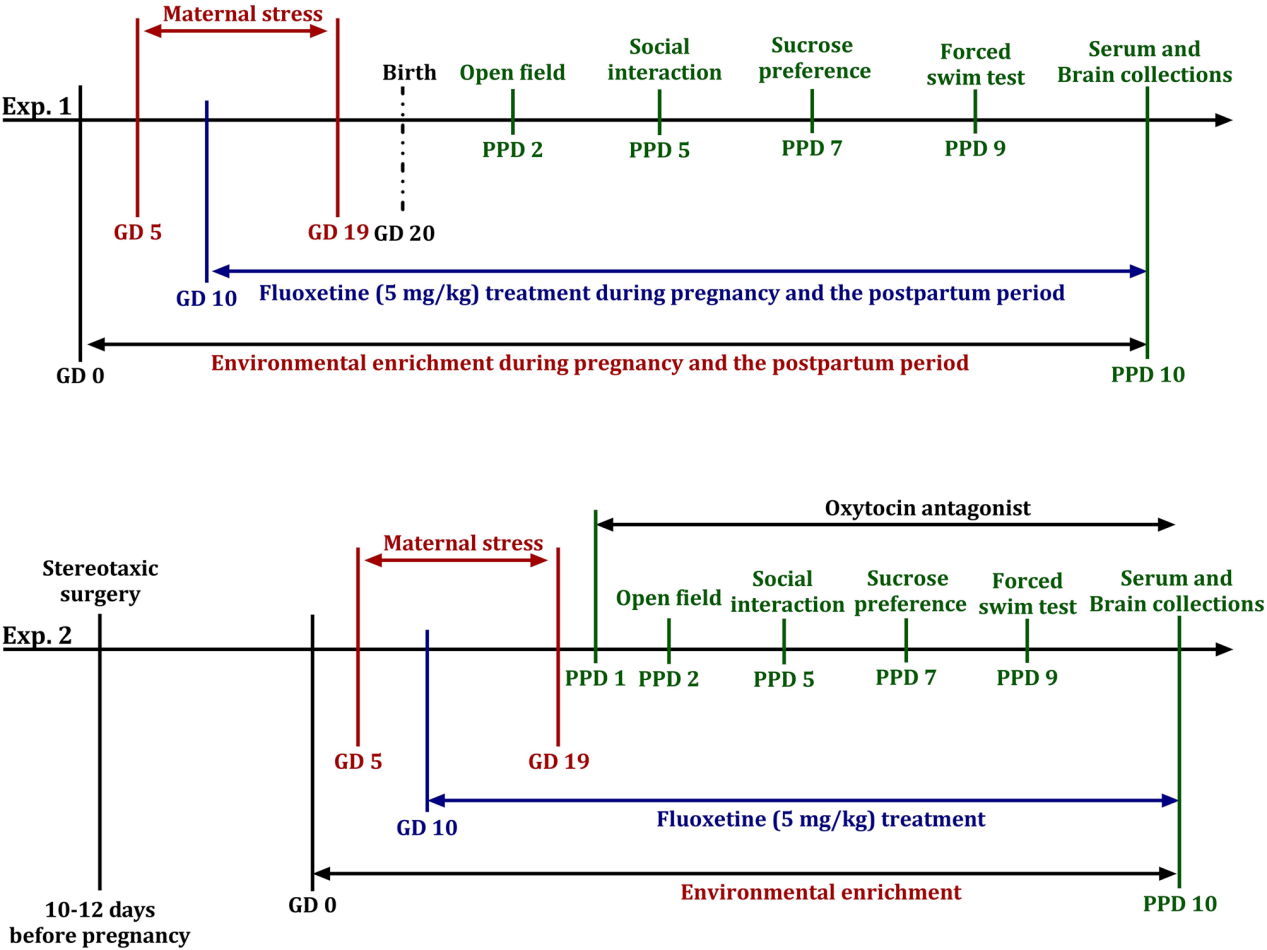
Experiment 1 tested the effects of gestational stress, fluoxetine treatment and environmental enrichment on anxiety (open field test), depression-like behavior (social interaction and sucrose preference, forced swim test), HPA-axis function (serum ACTH and corticosterone), and oxytocin in the brain during the postpartum period in dams. Experiment 2 investigated the FLX + EE mediated alleviation of the negative effects of gestational stress through blocking oxytocin receptor on anxiety and depression-like behavior, and HPA-axis function during the postpartum period in dams.

A schematic timeline of the experimental design is shown in Fig. 5. Experiment 1 investigated the combined effects of subthreshold FLX and non-significant EE (see explanation below) on postpartum anxiety and depression-like behavior, HPA axis function, and whole-brain OXT levels in gestational stressed dams. Pregnant mice were randomly divided into five groups (non-stress, stress, stress + FLX, stress + EE, and stress + FLX + EE). The mice were subjected to stress (stress) or left undisturbed (non-stress) during pregnancy (GD5 to GD19). In addition to stress, mice received FLX treatment (stress + FLX) in their drinking water (GD10 to PPD10), were housed in enriched cages (stress + EE; GD0 to PPD10) or were subjected to both FLX treatment and EE (stress + FLX + EE) during pregnancy. The FLX treatment period was selected based on the birth of serotonin neurons occurring at embryonic day 10.5 in mice^79^ and the correspondence of PPD10 in rodents to birth in humans^80^.

Experiment 2 investigated the role of OXT in the combined effects of subthreshold FLX and non-significant EE on gestational stress-induced changes in anxiety and depression-like behaviors, and HPA axis function, during the postpartum period in dams. To do so, OXT signaling was inhibited by blocking OXT receptors via brain infusion with an OXT receptor antagonist during the postpartum period. First, female mice underwent stereotaxic surgery 10–12 days before pregnancy (GD0). Then, pregnant mice were randomly divided into six groups (non-stress + vehicle, non-stress + OXT-A, stress + vehicle, stress + OXT-A, stress + FLX + EE + vehicle, and stress + FLX + EE + OXT-A). Pregnant mice were subjected to stress (GD5 to GD19) (stress), stress with a combination of FLX treatment (GD10 to PPD10) and EE (GD0 to PPD10) (stress + FLX + EE), or were left undisturbed (non-stress). During the postpartum period (PPD1 to PPD10) all groups received either an OXT antagonist (OXT-A) or vehicle (artificial cerebrospinal fluid; aCSF).

In both experiments, the dams were subjected to the open field-, social interaction-, sucrose preference-, and forced swim-test during the postpartum period. In experiment 1 and 2, serum adrenocorticotropic hormone (ACTH) and corticosterone were measured. In experiment 1, OXT levels in the brain were measured as well. In both experiment 1 and 2, ten animals were included in each group (n = 10/group).

### Environmental enrichment

Female mice were housed individually in enriched or standard environments during pregnancy and the postpartum period (GD0 to PPD 10)^81^. The enriched environment consisted of polycarbonate cages (58 × 38 × 20 cm) equipped with two plastic shelters, four tunnels and two cotton nestlets that were replaced once a week during cage cleaning with new objects of different shapes and colors. This enriched environment was selected based on our pilot studies that found no significant effects of EE on dams’ behaviors. The standard environment consisted of standard polycarbonate cages (27 × 21 × 14 cm) with only one cotton nestlet.

### Gestational restraint stress

Between GD 5 and 19, pregnant mice were exposed to three daily stress sessions. Briefly, dams were individually placed in plastic transparent cylinders (4 cm diameter, 10 cm long) under a single 60-W light bulb for 30 min as previously described^51^. There was a 3 h interval time between each session, the animals were exposed to the restraint stress between 10:00 (A.M.) and 7:00 (P.M.). Non-stressed dams were left undisturbed during pregnancy in their cages. We have previously shown that stress during these gestational days in female NMRI mice increases depression-related behaviors during the postpartum period^51^. To avoid litter effects on maternal anxiety and depression-like behaviors, all litters were culled to 8 pups (4 males and 4 females) per mother^82^.

### Maternal fluoxetine treatment

Five days after starting the restraint stress procedure, the pregnant mice were treated with FLX hydrochloride (TEMAD Co., Tehran, Iran; 5 mg/kg/day) during pregnancy and the postpartum period (GD10 to PPD 10) as previously described^51^. To minimize the unknown effects of repeated administration stress following drug treatment by invasive and stressful procedures resulting from repeated injections, osmotic minipumps or oral gavages, dams received FLX or vehicle (water) via regular drinking bottles. The FLX concentration was calculated at five-day intervals based on the average liquid consumption and body weight of the dams^51^. There was no other source for water except the drinking water containing FLX, therefore, dams were motivated by the thirst to drink the FLX solutions. The liquid consumptions of the vehicle-treated mice were evaluated every five days to assess possible impacts of the chronic FLX treatment on the liquid intake. No significant alterations were found between vehicle and FLX treated dams. The FLX dose was chosen based on our recent studies (Amani et al., under review) in which FLX did not influence HPA axis function, anxiety and depression-like behavior in postpartum dams.

### Stereotaxic surgery and OXT receptor blockade

In experiment 2, adult female mice underwent stereotactic surgery to enable brain infusions with OXT-A. Female mice were anesthetized by a combination of ketamine and xylazine (50 and 4 mg/kg respectively, Alfasan Co, Netherlands) and placed in a Stoelting stereotaxic instrument. Two single stainless-steel guide cannulae (26-gauge) were implanted into the left and right lateral ventricle (anteroposterior (AP): − 0.34 mm; mediolateral (ML): ± 1.0 mm; dorsal ventricular (DV): 2 mm;^83^. The guide cannulae were anchored to four micro stainless-steel screws using dental acrylic cement. A stainless-steel stylet (33-gauge) was inserted into each guide cannula to keep it free of debris and clogging. Female mice were allowed to recover from surgery for six days before breeding commenced. The left and right lateral ventricle were infused with OXT-A or vehicle (1 µl/mouse, 0.5 µl/side) by means of an internal cannula (33-gauge), terminating 1 mm below the tip of the guide, connected by polyethylene tubing to a 1-μl Hamilton syringe. The inner cannula was left in place for an additional 60 s to allow diffusion of the solution and to reduce the possibility of reflux^84^. OXT antagonist (OXT-A; (d(CH_2_)_5_^1^,Tyr(Me)^2^,Thr^4^,Orn^8^,des-Gly-NH_2_^9^)-Vasotocin, Bachem, Co) was dissolved in highly purified acetic acid artificial cerebrospinal fluid (aCSF) as described previously^85^. Postpartum dams received a daily intracerebroventricular infusion with a subthreshold dose of OXT-A (0.5 µg/1 µl/mouse) or vehicle (1 µl/mouse; aCSF) for 10 days (PPD1 to PPD 10). Behavioral testing or sampling in the dam occurred two hours after the injection. The location of infusion was checked post-mortem and no damage to other tissues than the track towards the targeted regions was found.

### Behavioral tests

To control for bias, behavioral measurements were carried out and observed by an expert observer blind to the treatment condition. All behavioral measurements were recorded using a stopwatch and performed during the light period (between 12:00 and 16:00 h) under illumination of 75 lx.

### Open field test

To assess anxiety-like behavior, the dams were subjected to the open field test on PPD 2 as previously described^86^. The apparatus consisted of a white box (40 × 40 × 20 cm) with one outer (10 × 10 cm; 12 squares) and inner (10 × 10 cm; 4 squares) zone. Each dam was placed in the middle of the apparatus and allowed to explore for 5 min. The time spent (s) in the inner zone was used as an index of anxiety-like behavior.

### Social interaction test

Social interaction was assessed on PPD 5 in dams as previously described^87^. Briefly, each dam was exposed to an unfamiliar same-treated dam (that was used in a parallel study). Animals were gently placed in opposite corners of a square (40 × 40 × 20 cm) arena and allowed to explore the apparatus for 7 min. The interaction between animals was defined as sniffing (the focal animal establishes contact or near-contact with its nose to the other animals’ body part), following (the focal animal moves/follow the other to maintain a close distance while the other animal is moving around/away), grooming (the focal grooming animal has one or both front paws on top of the other and pulls/licks at its fur), and climbing over each other (the focal animal climbs over the dorsal surface of the other animal)^88^. The total time interaction was measured as an index of social behavior.

#### Sucrose preference

The sucrose preference test was performed over on PPD 7 as described previously by our group^51^. The percentage of sucrose preference was considered as an anhedonia-like symptom. In short, after three days of acclimation to the two-bottle choice paradigm, each animal was given two bottles over a 48-h period: one containing a 2% sucrose solution or 2% sucrose solution + FLX (A) and the other containing tap water or water + FLX (B). The position of the bottles A and B was changed every 12 h to avoid side bias. The sucrose preference was calculated as the percentage of sucrose solution ingested relative to the total amount of liquid consumed.

#### Forced swim test

To assess behavioral despair, dams were subjected to the forced swim test on PPD 9. Immobility time was considered as a state of behavioral despair^82^. In short, dams were placed individually in transparent glass cylinders (height: 25 cm, diameter: 10 cm, depth: 15 cm) filled with fresh water (25 ± 1 °C) and the total duration of immobility was recorded for 6 min, of which the last 4 were analyzed. Each dam was considered immobile when it ceased struggling and remained floating motionless in the water, making only those movements necessary to keep its head above water.

#### Enzyme-linked immunosorbent assay (ELISA)

On PPD 10, the dams were anesthetized with pentobarbital (60 mg/kg, ip; between 12:00 and 16:00 h), blood and brain tissue were collected as previously described^51^. Briefly, trunk blood was collected into sterile 2 ml microtubes, allowed to clot on ice-packs (15 min), and centrifuged at 3000 rpm for 10 min to detect serum. After collecting the blood, mice were perfused with ice-cold NaCl, 0.9% to prevent the retention of blood in the brain. Afterwards, whole brain was rapidly removed, snap frozen in liquid nitrogen, and placed into 2 ml microtubes. All serum and brain samples were stored at − 80 °C until further processing. ACTH (Abcam Co, ab263880; sensitivity = 6 pg/ml) and corticosterone (Mybiosource, MBS264846; sensitivity = 1 ng/ml) were measured in serum, and OXT (Mybiosource, MBS745701; sensitivity = 1 pg/ml) was measured in brain tissue using specific quantitative sandwich ELISA kits according to the manufacturer’s instructions. All samples and standards were assayed in duplicate. To obtain brain tissue supernatant, whole brain tissue was added to 1000 μl homogenizing buffer including TBS plus 0.2% Triton X-100, 2 mM EDTA, PBS 1 mM PMSF, and protease inhibitors, and then centrifuged at 15,000 g for 15 min at 4 °C^82^. The total protein levels were also determined by the bicinchoninic acid assay kit (Sigma Co, BCA1). The concentration of OXT is presented as pg/mg protein. Whole-brain analysis was performed since neuropathologies underlying brain disorders are dispersed over the brain, as evidenced by the involvement of various large-scale brain networks in depression in humans^89^ and susceptibility to depression-like behavior in rodents^82,90,91^.

### Data analysis

One-way analysis of variance (ANOVA; IBM-SPSS software-26) was used to analyze the data (no data were excluded). For each behavior test p-value was corrected for multiple testing (adjusted p value) using the Benjamini–Hochberg method^92^. Further analysis was carried out using Tukey's HSD post hoc tests for multiple comparisons. All data are presented as the mean + standard error of the mean (SEM). A *p*-value < 0.05 was considered statistically significant.
